# Epidemiology of coronary artery disease and stroke and associated risk factors in Gaza community –Palestine

**DOI:** 10.1371/journal.pone.0211131

**Published:** 2019-01-25

**Authors:** Amal Jamee Shahwan, Yehia Abed, Ileana Desormais, Julien Magne, Pierre Marie Preux, Victor Aboyans, Philippe Lacroix

**Affiliations:** 1 INSERM UMR 1094, Tropical Neuroepidemiology, Limoges, France; 2 University of Limoges, School of Medicine, Institute of Neuroepidemiology and Tropical Neurology, CNRS FR 3503 GEIST, Limoges, France; 3 Cardiology department, Ministry of health, Gaza-Palestine; 4 University Al Quods, Gaza, Palestine; 5 Department of Thoracic and Vascular Surgery–Vascular Medicine, Dupuytren University Hospital, Limoges, France; 6 Department of Cardiology, Dupuytren University Hospital, Limoges, France; Clemson University, UNITED STATES

## Abstract

**Aim of study:**

To determine the prevalence of cardiovascular disease and associated risk factors in the population of Gaza strip in Palestine.

**Methods:**

A cross-sectional stratified cluster sample design was applied in this study. A sample of 2240 participant (1121 males and 1119 females) aged ≥25 years participated in the study. For each individual, trained staff administered a questionnaire, where all variables of interest followed WHO’s STEP wise approach to surveillance chronic disease risk factors (STEPS) (WHO, 2001). Sociodemographic data, anthropometric measure (body mass index, blood pressure), and biochemical test (blood sugar and lipids profiles) were measured. Short International Physical Activity (IPAQ) questionnaire form was used. Bivariate analysis and logistic regression were used with SPSS (version 22.0) to analyze the data.

**Results:**

The most common condition was coronary artery disease (8.3%), followed by stroke events (3%). The associated risk factors were obesity (47.8%), hypertension (28.4%), current smoking account for (23.2%), diabetes mellitus (19.1%), high cholesterol level (8.8%), and high triglycerides level (40.2%). Additionally, the proportion of being physical active was found to be low (48.3%); particularly with increasing age. More than 30% of the population has less than 4 days of consumption of fruit and vegetables per week and 65.9% has less than 2 servings per day.

**Conclusion:**

The burden of CVDs and their associated risk factors is considerable in Gaza and represents a major public health concern. Effective strategies in management, education and healthcare centers are required for an accurate management and implementation of preventive measure in this area.

## Introduction

In the previous years, there were dramatic changes in the occurrence of the major manifestations of cardiovascular disease (CVDs), mainly coronary artery disease (CAD) as well as cerebrovascular disease (CBVD). Cardiovascular diseases are now recognized as the leading cause of death and disability worldwide [1]. In 2015, it was estimated that 17.7 million people died from CVD worldwide, representing 31% of all global deaths; out of whom, 7.4 million were due to CAD, and 6.7 million were due to stroke [2]. In the United States (US), 92.1 million adults experienced at least one type of CVD. By 2030, 43.9% of the US adult population is projected to have some form of CVD. However from 2004 to 2014, death rates attributable to CVD declined by 25.3% in that country [3]. Three quarters of global CVD deaths occur in Low and Middle-Income Countries (LMIC). In 2015, among 17 million premature deaths (under the age of 70) was due to Non-Communicable diseases (NCDs); 82% of them took place in LMIC and 37% of the deaths are caused by CVDs, almost equal in males and females [4–6]. Arab countries in the Middle East have undergone rapid and dramatic socioeconomic changes. In these countries which have young populations, CVD mortality accounts for 45% of deaths [7]. The rates of CVDs deaths were up to 42%,38%,32% and 23% respectively in Saudi Arabia, the United Arabic Emirates, Bahrain and Qatar [8].

Similar to other countries, an epidemiological transition occurred in Palestine. The leading causes of death in the Palestinian community are NCDs, up to 50% of all deaths. The incidence is higher in the West Bank (WB) 57% vs 40% in Gaza Strip (GS) [9]. In 2014, CVD was reported as the first cause of deaths in Palestine accounting for 29.5%, CBVD was the third common cause corresponding to 11% of all deaths [10]. Most data are from hospital based, few studies were conducted on prevalence of CVD risk factors in Gazans population. Data regarding the prevalence or incidence of CAD and CBVD, in this community are lacking.

The aim of this study is to document the prevalence of CVDs (i.e. CAD, Stroke) and associated risk factors in Gazan community.

## Methodology

The Gaza strip is a small 365 square kilometers area of Palestinian occupied territories; it is a very crowded place as around 1.9 million Palestinians live in. Gaza strip consists of 5 governorates with fourteen villages and eight refugee camps [11]. We conducted a cross-sectional study using stratified cluster sample, with the advantage of covering a wide geographic area, in 5 governorates spanning both urban and rural spaces between July and October 2017. The target population include 673 523 inhabitants, almost 35.8% of the total Gaza strip population.

The sample size was 2240 calculated by using Epi-info (epidemiological information, statistical program, v 3.1.1, CDC,2015), considering a CVDs prevalence among US adult Americans population (36.6%) according to American Heart Association statistical update [3]. with a precision of 2% as error level, and 95% confidential interval, and a cluster effect of ‘1. The number of individuals in the sample was proportional to the number of the population in each district. The cluster areas were randomly chosen and each area yielded 15–20 households. The investigators went from door to door, and in every house, all adults aged ≥25 years were proposed to participate. Pregnant women and individuals with mental disability were excluded.

The study was conducted in accordance with WHO’s STEP wise approach to surveillance chronic disease risk factors (STEPS, WHO 2001) which involves three primary “steps”. At step 1, we used questionnaire to assess demographic, socioeconomic, reported behavioral, and lifestyle risk factors for chronic diseases. In addition, short international physical activity (IPAQ) form questionnaire was used. At Step 2, blood pressure on both arms and anthropometrical parameters were collected: height, weight, waist circumference, and Heart rate, by pulse oximeter, were measured. Then at Step 3, 2226 participants underwent a venous puncture for lipids analysis, fasting or random blood sugar (DIAVUE Prudential). Fourteen participants refused.

### Variables definitions

Height was measured with a wall-mounted stadiometer to the nearest 0.1 cm. Weight measured to the nearest 10 grams with electronic scales (Seca, Hamburg, Germany). Waist circumflex measured mid-way between the lateral lower rib margin, and the iliac crest and according to European data which was used for Eastern Mediterranean population, we defined normal waist <80 cm in females and < 94 cm in males [12]. Diabetes mellitus(DM) was defined as capillary blood sugar level ≥126 mg/dl if the participant was fasting or ≥ 200mg/dl if the participant was non-fasting and or self-reported as currently taking any diabetes medications [13]. For hypertension (HTN), we considered subjects to have HTN if their average systolic blood pressure (SBP) in both arms was ≥140 mmHg or their average diastolic blood pressure (DBP) ≥90 mmHg, or if they were being treated for HTN [14,15]. Body mass index (BMI) was calculated as weight in kilograms divided by their height in meters in squared and considered anyone with a BMI of 25kg/m^2^ or higher overweight, and more than 30kg/m^2^ as obese according (WHO classification). Low density lipoprotein (LDL cholesterol) was calculated using modified Friedewald equation [16]. Lipids profiles abnormalities were classified according to ATPIII Guidelines as total cholesterol levels ≥ 240mg/dl, high density lipoprotein (HDL cholesterol) ≤40 mg/dl in men and ≤50 mg/dl in women, low density lipoprotein(LDL cholesterol) ≥ 160 mg/dl, and triglycerides ≥150 mg/dl [17]. CAD was defined in our study by self-reported history of hospitalization for angina pectoris, myocardial infarction, procedures performing percutaneous coronary intervention or coronary bypass graft and has been check by their medical prescription list. Stroke was identified with the question that raises the existence, among the house inhabitants of a person having a history of stroke diagnosed by a physician (we asked “have you ever been told by a physician that you suffered a stroke”?).

### Authorizations

The study protocol was approved by the Ethical of Human Research Committee of Palestinian Health Research Council, and an individual written or verbal consent was obtained by each participant.

### Data analysis

Data was analyzed using Statistical Package for Social Sciences version 22. Categorical variables are presented as percentage, and continuous data as the means and standard deviation. We first compared differences in socio-demographic as well as lifestyle-related factors using chi square test, Somers’ D test (for ordinal qualitative variables), student’s t-test, or Wilcoxon’s rank sum test as appropriate. Then we used logistic regression to examine the association between CVDs and their risk factors with/without adjustment for covariates. Any covariate with a p-value ≤ 0.25 in the age-and gender-adjusted model was considered in multiple logistic regression model. The final model was obtained using a backward stepwise procedure. We examine confounding at each step and then we examine first-order interactions in the final model. Statistical significance level was set-up at 0.05 for all analyses.

## Results

A total of 2240 participant were included into the study. Sociodemographic profiles and the burden of Cardiovascular risk factors of the participants are displayed in “Table 1”. No significant difference was seen according to age group distribution and gender. The mean age of study population is 47.4 years (47.1 in male and 47.8 in female). Forty percent of participants lived in refugee camps. Furthermore, 7% of the females did not have any formal schooling, while 25.8% of the study population completed university education, and 7.6% achieved postgraduate degree. The monthly income was less than $150 for 36.8% of the participants. Regarding CVD risk factors hypertension and diabetes were higher among females. BMI was ≥ 30kg/m^2^ in 47.8% of the cases. Obesity was more common in females than males, 60.2%, 35.5% respectively. Conversely The overweight was more common in males than females 39.1% vs 26.7% (*p* value <0.001). In addition, 8.8% and 40.2% of study population had high levels of total cholesterol and triglycerides respectively.

**Table 1:** Socio- demographic characteristics and burden of cardiovascular risk factors of study population.

Variables	Gender						P value*
	Total		Males		Females		
	No.	%	No.	%	No.	%	
Age group							
25–34 years	503	22.5	277	24.7	226	20.2	0.061
35–44 years	494	22.1	226	20.2	268	23.9	
45–54 years	528	23.6	266	23.7	262	23.4	
55–64 years	420	18.8	205	18.3	215	19.2	
65+ years	295	13.2	147	13.1	148	13.2	
Locality							
Urban	798	35.6	373	33.7	420	37.5	0.002
Rural	529	23.6	245	21.9	284	25.4	
Camp	913	40.8	498	44.4	41%	37.1	
Education							
No formal schooling	106	4.7	28	2.5	78	7.0	<0.001
Primary schooling	238	10.6	114	10.2	124	11.1	
University complete	579	25.8	335	29.9	244	21.8	
Post graduate	170	7.6	118	10.5	52	4.6	
Income in US ($) per month							
<$150	825	36.8	373	33.3	452	40.4	0.001
$150–500	944	42.1	478	42.6	466	41.6	
>$500	471	21.0	270	24.1	201	18.0	
Cardiovascular risk factors							
Hypertension	636	28.4	292	26.0	344	30.7	0.014
Diabetes	427	19.1	206	18.4	221	19.7	0.408
overweight	737	32.9	438	39.1	299	26.7	<0.001
obesity	1070	47.8	397	35.5	673	60.2	
Lipids profiles							
Total cholesterol ≥240 mg/dl	195	8.8	65	6.1	127	11.4	<0.001
LDL cholesterol ≥160 mg/dl	187	8.4	71	6.4	116	10.3	0.001
Low HDL cholesterol mg/dl	1569	70	779	69.5	790	70.6	0.847
Triglycerides ≥150mg/dl	895	40.2	480	43.1	415	37.3	0.006

Overweight: BMI (25–29.9 kg/m2)

Obese: BMI (≥30 kg/m2)

A Chi-square test or Somers’ D test (for ordinal variables) was used.

Data on the lifestyle related risk factors are displayed in “Table 2”. Smoking was by far less frequent among women since smoking is not accepted for women in Gaza. The prevalence of smoking among males increased with age until the age of 44 and decreased later. Additionally, 64.3% of men in age group 25–34 years started to smoke before the age of 18 years. The consumption and serving of fruits and vegetables was low, with minor differences in gender and age groups.

**Table 2:** Life style-related risk factors in the study population by age for men and women.

Variables	Gender	Age groups in years %					Overall %	Age Adjusted %	P value
		25–34	35–44	45–54	55–64	65+			
Current Smoking	All	30.2	23.9	22.3	22.4	12.9	23.2	23.6	<0.001
	Males	50.5	50.4	42.9	42.4	25.9	44.0	44.5	<0.001
	Females	5.3	1.5	1.5	3.3	0.0	2.4	2.61	<0.001
Age of onset of smoking <18y	All	61.2	50.0	53.4	47.9	44.7	53.3	52.7	0.05
	Males	64.3	49.1	55.3	48.3	44.7	54.4	53.8	0.01
	Females	25.3	75.0	0.0	42.0	0.0	33.3	31.3	0.15
Low Physical Activity	All	36.4	45.1	49.4	55.2	61.7	48.3	47.3	<0.001
	Males	27.1	37.2	40.6	42.9	50.3	38.3	37.7	<0.001
	Females	47.8	51.9	58.4	67.0	73.0	58.3	57.1	<0.001
Fruits+ vegetable intake < 4days /week	All	26.9	34.0	33.8	28.3	29.2	30.7	30.5	<0.001
	Males	24.6	32.0	31.3	26.3	26.5	28.3	28.3	0.04
	Females	29.6	35.7	36.4	30.2	31.8	33.1	32.8	0.29
Fruits+ vegetable <2 serving / day	All	62.7	70.1	65.6	68.3	61.4	65.9	65.7	0.03
	Males	62.0	64.0	63.0	65.0	59.9	63.1	62.8	0.64
	Females	63.6	74.8	68.2	70.7	62.8	68.6	68.2	0.07

Data are represented as percent (%)

A Chi-square test was used to compare the difference in risk factors for CAD and stroke across age subgroups for the overall population as well as for each gender.

Overall 218 subjects (9.7%) of participants had CVD 11.5%in males and 8.0% in females “Table 3”. CAD was present in 185 persons 8.3% (95% CI, 7.14%-9.46%), males reported a higher prevalence (10.1%) than females (6.4%). Among the 2240 participants we found only 67 cases which reported a history of stroke 3% (95%CI, 2.28%-3.72%) with no difference in gender (3.5% vs.2.5%). The clinical disorders were limited to one territory for 184 participants (8.2%) involving 9.5% of the males and 7.0% of the females. Two territories were involved in 34 participants (1.5%), with a1.5 higher risk in males.

**Table 3:** Prevalence of cardiovascular disease (CAD, stroke) by gender.

Cardiovascular diseases	Gender							
	All		Males		Females		Odds Ratio (95%CI)	P value
	No.	%	No.	%	No.	%		
CAD	185	8.3	113	10.1	72	6.4	1.63 (1.19–2.21)	0.002
Stroke	67	3.0	39	3.5	28	2.5	1.40 (0.86–2.29)	0.175
CAD or Stroke	184	8.2	106	9.5	78	7.0	1.41 (1.05–1.91)	0.037
CAD and Stroke	34	1.5	23	2.1	11	1.0	2.17 (1.05–4.47)	0.030
CVD	218	9.7	129	11.5	89	8.0	1.51 (1.13–2.01)	0.005

Female gender is the reference group, CAD: Coronary artery disease, CVD: cardiovascular disease

Odds Ratio: male vs female CI: confidence interval 95%

Fisher’s exact method was used

After adjustment by age and gender, the most important risk factors associated with CVD were HTN, and DM, *p v*alue <0. 001 “Table 4”. Hypertension was associated with a 3-fold increased risk of CVD and diabetes mellitus a 2.5-fold.

**Table 4:** Multivariate logistic regression (adjusted for age, and gender) with CVD as dependent variable and major risk factors as independent variables.

Variables	Univariate analysis		Adjusted analysis	
	Odds Ratio (CI)	P value	Odds Ratio (CI)	P value
Age	0.94 (0.93–0.95)	<0.001		
Gender	1.51 (1.13–2.01)	0.005		
Hypertension	4.73 (3.53–6.31)	<0.001	2.51 (1.81–3.50)	<0.001
Diabetes	4.49 (3.36–6.01)	<0.001	2.22 (1.61–3.11)	<0.001
BMI≥30kg/m^2^	1.44 (1.10–1.91)	0.011		
Current smoking	1.07(0.77–1.48)	0.686		
High cholesterol	1.27(0.80–2.01)	0.303		
High triglycerides	1.43 (1.08–1.89)	0.013		

BMI: Body mass index

Logistic regression model was used

Odds Ratio: male vs female. CI: confidence interval (95%)

## Discussion

Our study is the first to report the prevalence of CVDs among Gazans in Palestine. Previous surveys were hospital based or on United Nation Relief and Work Agency (UNRWA) report. The prevalence of CVDs nears 10% of the population above the age of 25years in this area.

Data on the CVD epidemiology in the Middle-East are limited. In the Coronary Artery Disease in Saudi study (CADISS), a national community-based study conducted in urban and rural area of Saudi Arabia; 17 232 subjects aged 30–70 years randomly selected were included. Coronary artery disease was diagnosed on positive questionnaire of angina or history of possible myocardial infarction. The prevalence of CAD was 5.5% (6.6% in males and 4.4% in females) [18]. Similar findings have been reported in a Lebanese cross-sectional study using multistage cluster sample including 1200 subjects ≥40 years of age. The prevalence of CAD was 13.4% (17.8% in males, 9.0% in females) [19]. In the Jordanian population, the prevalence of myocardial infarction was reported at 5.9% in adults over 40 years [20]. Regarding stroke our results are in line with another Lebanese study including1515 individuals with a mean age of 57.2 years; stroke and or transit ischemic attack were (3.6%), and the prevalence of any stroke symptom was up to 12.1% [21]. According to a systemic review in Middle east, the prevalence of stroke was estimated between 508 to 777 per 100,000 population [22], more commonly in males than females [22,23]. Two hospitals based studies conducted in Palestine, showed more stroke cases among females than males groups [24,25].

Number of risk factors were identified in epidemiological surveys. In 2016 the global burden disease (GBD) for risk profiles in Middle east and north Africa (MENA) stranded out these risk factors by order of penetrance, high blood pressure ranked as the first, followed by obesity, diabetes then smoker [26]. The same grading was retained for Palestine [26].The UNRWA 2016 report for Gaza, found 20.1% subjects with hypertension among population aged ≥ 40 years (Palestinian Refugees of Syria included) [27]; we reported a prevalence of (28.4%). The literature review revealed a higher prevalence of hypertension in Arab countries 29.5%. The same prevalence was found in the US (29.6%), and (30%) in United Kingdom [28].

Obesity is a common health problem worldwide particularly in Arab world. The prevalence of obesity and overweight in Arab states ranges from (25% to 81.9%) [29]. The Global burden disease (GBD) 2016 estimated that high BMI was the leading six risk factors in Arab countries [26]. The overall obesity in our study was found to be 47.8%, with higher prevalence in females (60.2%), the mean BMI was 30.35. It is in line with previous study performed in urban Palestinian population of Ramallah, describing an obesity prevalence up to 41% (49% and 30%) in females and males respectively [30], these results were higher than those in the US population (36.5%)[31] or in Israel which the National Health and Nutrition 1999–2001 (MABAT) was conducted among 2782 persons, and showed a prevalence of overweight in 39.3% and obesity in 22.9% adults population. The Israeli Arab population was more obese than the Jewish one [32]. Also, our prevalence was higher than among Tunisian males and females (18.2%, 33.5%) respectively [33].

Diabetes mellitus was identified in our study with a prevalence of 19.1%. WHO report 2015 from Middle East Region (Bahrain, Kuwait, Oman, and the United Arab Emirates) estimated that the prevalence of DM was between 3.5% and 30% [34]. In a study in Saudi Arabia, the prevalence of type 2 diabetes among 2355 adults was 29.3% [35]. For smokers, our estimated prevalence was 23.2%. The WHO report on the global tobacco epidemiology (2011) estimated that Europe had the highest prevalence of smokers (35%); and the lowest was in Africa. The global Adult tobacco survey showed that in fourteen LMIC, 48.6% of men and 11.3% of women were tobacco users [36]. Additionally, in our neighbour countries, a cross sectional study conducted in Lebanon, among 2836 adults ≥18 years of age, showed that the current smoking prevalence rate was 34.7% with higher rate in males than females,42.9% and 27.5% respectively [37]. In the BREATH study, 62 086 subjects aged ≥ 40years in ten countries in MENA region, were interviewed regarding smoking habits, with equal number of men and women. The smoking rate was estimated to be 31.2%, ranging from 15.3% in Morocco to 53.9% in Lebanon. The proportion was higher in men (48.0%) than in women (13.8%) [38], the prevalence of female smokers has traditionally been low due to the Eastern Mediterranean region’s conservative cultural and social values. Less than one third of our study population have dyslipidaemia. However, our finding was counterpart with data from ACE (Cardiovascular Epidemiological) study in United Arab Emirates, the prevalence was 74% [39], while the high prevalence of dyslipidaemia in our study in young adults (age 25-34years) calls for an earlier screening for dyslipidaemia and other risk factors. Another important factor observed in our study was the proportion of people with low physical activity, it was estimated that half of the population 48.3% had low physical activity. Women exercised less than men (58.3%). We ascribe these phenomena to the fact that women have maternal responsibility and were more obese. In a study for 163 556 persons in 38 Muslim countries who completed IPAQ, the total physical inactivity prevalence was 32.8%. The prevalence among Arab countries was 43.7% *vs* 28.6% in non-Arab countries, and Arab women were more physically inactive [40]. In a cross-sectional study conducted in Saudi Arabia, physical activity was assessed using the global physical activity questionnaire among 4758 participants. The prevalence of low activity was 66.6% (60.1% in males, and 72.9% in females). Similar data were found in the Arabian Gulf region [41,42].

Nutrition is an important determinant of health; inadequate consumption of fruit and vegetable is a factor that can play role in morbidity. As other studies in Arab gulf countries our study population did not consume sufficient quantities of fruits and vegetables. A study analyzed data from 197 373 adult participants from 52 countries taking part in the world health survey in LIMIC (2002–2003). The prevalence of low consumption of fruit and vegetable was 77.6% in males and 78.4% in females [43].

## Strengths and limitations

There are few limitations to our study. First, the cross-sectional nature of this study design limits the interference of causal relation-ships between risk factors and CAD or stroke. Also, the prevalence of high blood pressure and raised blood glucose have been over estimated because these two risk factors were evaluated once (no reevaluation during another visit, and no three consecutive measures of BP according to WHO step). Further, possible bias could have been introduced since study was conducted at home and data concerning more risk factors and history of CAD and stroke were self- reported. Also, the wide age range of participants, 25 years and more is both a strength and a weakness of this study. However, 32% of the population was aged 55 years and more, who have a greatest prevalence of CVD and risk factors. Even so we found considerable charge of risk factors in young population. Also, this study has strengths points: women were well represented, with a male: female ratio of 1, and it was performed in a large mixed area with representative sample, and good response rate; making it the first study in Palestine to report a national estimate for CVD prevalence.

## Conclusion

This study was the first nationwide endeavor that provides information about the prevalence of CVDs and the level of cardio vascular risk factors among palestinian community in Gaza.

A rate of CVD has been reported in our population, 10% reflecting a serious health problem in Gaza strip. Obesity, hypertension and diabetes, were highly prevalent. Increased, effort and research to monitor and improve strategies and policies for reducing cardiovascular risk are mandatory.

## Supporting information

S1 Dataset Database of study participants. (XLSX)

S1 Text Definition of study variables. (DOCX)
