# Supplementary material for: Epidemiology of coronary artery disease and stroke and associated risk factors in Gaza community –Palestine
Source: PLoS One. 2019 Jan 25;14(1):e0211131. doi: 10.1371/journal.pone.0211131 (PMC6347168; doi:10.1371/journal.pone.0211131)
Supplement: S1 Text — (DOCX) [file pone.0211131.s002.docx]

**Variable codes for the study**

**Survey Information**

D1: Participant Identification Number

D1: Date:( mm/yyyy)

S1: Consent: (1.yes/ 2. No)

S2: Area Name (1. Urban/ 2. rural/ 3. Camp)

==========================================================

**Demographic Information**

D1: Gender: (1. Male /2. Female)

D2: Age group

1. 25years-35years
2. 2.35years-45years
3. 3.45years-54years
4. 55years-64years
5. ≥65years

D3: Education:

1. No formal Schooling (0 years
2. Primary School (0-6years)
3. Secondary School (7-9 years)
4. High School (10-12 years)
5. University completed (12-15 years)
6. Post graduate degree (>15 years)

D4: Income per month ($)

1. <150$
2. 150$-500$
3. >500$

======================================================================

**Steps 1: Behavioral Measurement**

Bm1: Smoking: (1.yes / 2. No)

Bm2: Age of onset of smoking:(1:<18 years/2. ≥18 years)

Bm3: Consummation of fruit and vegetable per week: (1. <4 times/2. ≥4 times)

Bm4: Number of fruit and vegetable serving per day: (1. <2 times/2. ≥2 times)

Bm5: Physical activity:(0. Low/1.moderate/2.high)

Bm6: History of hypertension: (1.yes/2. No)

Bm7: History of Diabetes: (1.yes/2. No)

Bm8: History of stroke (1.yes/2. No)

Bm9: History of coronary artery disease (1.yes/2. No)

Bm10: Cardio vascular disease: (1.yes/2. No)

Bm11: Type of cardiovascular disease: (0. No /1.1CVD/ 2. 2CVD)

======================================================================

**STEP2: Physical measurement**

Pm1: Body mass index group:

1. < 18.5 kg/m^2^
2. 18.5-24.99 kg/m^2^
3. 25-29.99 kg/m^2^
4. ≥30 kg/m^2^

Pm2: Systolic blood pressure

Pm3: Diastolic blood pressure

======================================================================

**STEP2: Biochemical measurement**

Cm0: Random blood glucose:0<200 mg/dl / 1. ≥200 mg/dl

Cm0’: Fasting blood glucose:0. <126 mg/dl / 1. ≥126 mg/dl

Cm1: Cholesterol 1. ≥240mg/dl/ 2. <240 mg/dl

Cm2 HDL cholesterol (1. Low/ 2. Normal)

Cm3:LDLcholesterl1 ≥160 mg/dl 2. <160 mg/dl

Cm4:Triglyceride1≥150 mg/dl 2. <150 mg/dl

======================================================================
